# Corrigendum: Controlled growth of CH_3_NH_3_PbI_3_ nanowires in arrays of open nanofluidic channels

**DOI:** 10.1038/srep21751

**Published:** 2016-02-25

**Authors:** Massimo Spina, Eric Bonvin, Andrzej Sienkiewicz, Bálint Náfrádi, László Forró, Endre Horváth

Scientific Reports 6: Article number: 1983410.1038/srep19834; published online: 01252016; updated: 02252016.

Bálint Náfrádi was omitted from the author list in the original version of this Article. This has been corrected in the PDF and HTML versions of the Article.

The Author Contributions section now reads:

L.F. initiated the research. E.H. synthesized the perovskite solutions and discovered the graphoepitaxial nanowire growth. M.S. and E.B. prepared the microfabricated devices. M.S., E.B. and B.N. performed the photocurrent measurements and analyzed the data. A.S. performed and analyzed the fluorescence measurements. E.H., A.S., M.S., E.B., B.N. and L.F. discussed the results and implications and commented on the manuscript at all stages.

