# Supplementary material for: Controlled growth of CH3NH3PbI3 nanowires in arrays of open nanofluidic channels
Source: Sci Rep. 2016 Jan 25;6:19834. doi: 10.1038/srep19834 (PMC4726236; doi:10.1038/srep19834)
Supplement: Supplementary Information [file srep19834-s1.pdf]

Supplementary Materials:

Controlled growth of $CH_3NH_3PbI_3$ nanowires in arrays of open nanofluidic channels

Authors: Massimo Spina[1], Eric Bonvin[1], Andrzej Sienkiewicz[1,2], László Forró[1], Endre Horváth[1,*]

Affiliations:

[1]Laboratory of Physics of Complex Matter (LPMC), Ecole Polytechnique Fédérale de Lausanne, 1015 Lausanne, Switzerland

[2]ADSresonances, CH-1028, Préverenges, Switzerland

Materials and Methods

Preparation of $MAPbI_3$ solution

$MAPbI_3$ single crystals and polycrystalline powder were prepared by precipitation from a concentrated aqueous solution of hydriodic acid (57 w% in H_2O, 99.99 % Sigma-Aldrich) containing lead (II) acetate trihydrate (99.999 %, Acros Organics) and a respective amount of $CH_3NH_3^+$ solution (40 w% in H_2O, Sigma-Aldrich). A constant 55-42 ^oC temperature gradient was applied to induce the saturation of the solute at the low temperature part of the solution. Besides the formation of hundreds of submillimeter sized crystallites several large $MAPbI_3$ crystals with 3x5 mm silver-grey mirror-like facets were grown after 21 days. The saturated solution (≈50 w%) of $MAPbI_3$ was prepared by dissolving the as-synthetized sub-millimeter

sized single crystals as well as the polycrystalline powder in 550 µl of dimethylformamide (DMF, Sigma-Aldrich).

Microchannel fabrication

A high resolution positive e-beam resist (ZEP520A) was spin-coated on the target substrate (Si, SiO_2-thermal evaporated, TiO_2, SiO_2-quartz) that was previously heated for 5 min at 180 °C. The sample was subsequently baked for 5 min at 180°C. The nanofluidic channels were patterned by e-beam lithography with a Vistec EBPG5000 operating at 100 kV and 1 nA, with a dose of 300 μCcm^{-2}. After exposure the resist was developed for 1 min in Amyl-Acetate, rinsed in a solution of MiBK:IPA (90:10) and dried with a nitrogen blow gun. Well-defined nanofluidic channels on the Si and SiO_2 substrate were fabricated by reactive ion etching with an Adixen AMS200 and a gas mixture of Ar and C_4F_8.

Graphene growth

Graphene was prepared on copper foil via a Chemical Vapor Deposition (CVD) method. Details of the synthesis, the transfer process and micro-texturing by e-beam lithography can be found in our recent work (*11*).

Nanofluidic-assisted perovskite nanowire synthesis

1 µl of saturated solution was dropped on top of the nanofluidic channels. Capillary forces drove the liquid inside the channel. After the solvent evaporation, the substrate was heated at 80 °C for 60 seconds in order to facilitate the crystallization of the nanowires. The sample was then immersed in chloroform for 1 min to remove the ZEP520A resist, leaving the elongated crystallites of $MAPbI_3$ in the predefined locations.

The same procedure was applied to grow $MAPbI_3$ nanowires in well-defined nanofluidic channels on the Si and SiO_2 substrate

SEM characterization

Scanning Electron Microscope images were acquired with a MERLIN Zeiss electron microscope. The samples were held at a working distance of 3-4 mm and images were taken with an accelerating voltage of 3 kV and a beam current of 73 pA.

Optical Imaging

Videos of the nanowires growth and corresponding images were taken with a DSLR camera body (type Canon EOS 5D Mark III) mounted to an optical microscope (Olympus BL-2) with a magnification of 80x.

Optical microscopy and photoluminescence measurements

Optical microscopy images of perovskite nanowire bundles as well their corresponding photoluminescence spectra were obtained using a customized inverted biological epi-fluorescent microscope (TC5500, Meiji Techno, Japan), which was combined with a commercially-available spectrofluorometer (USB 2000+XR, Ocean Optics Inc., USA). This setup enabled us to simultaneously record microscopic images and acquire photoluminescence spectra of $MAPbI_3$ deposits on glass microscope slides under visible light illumination, as well as under excitation with the monochromatic incoherent light at 470 nm and 546 nm wavelengths.

SEM characterization of micro-fabricated periodic array

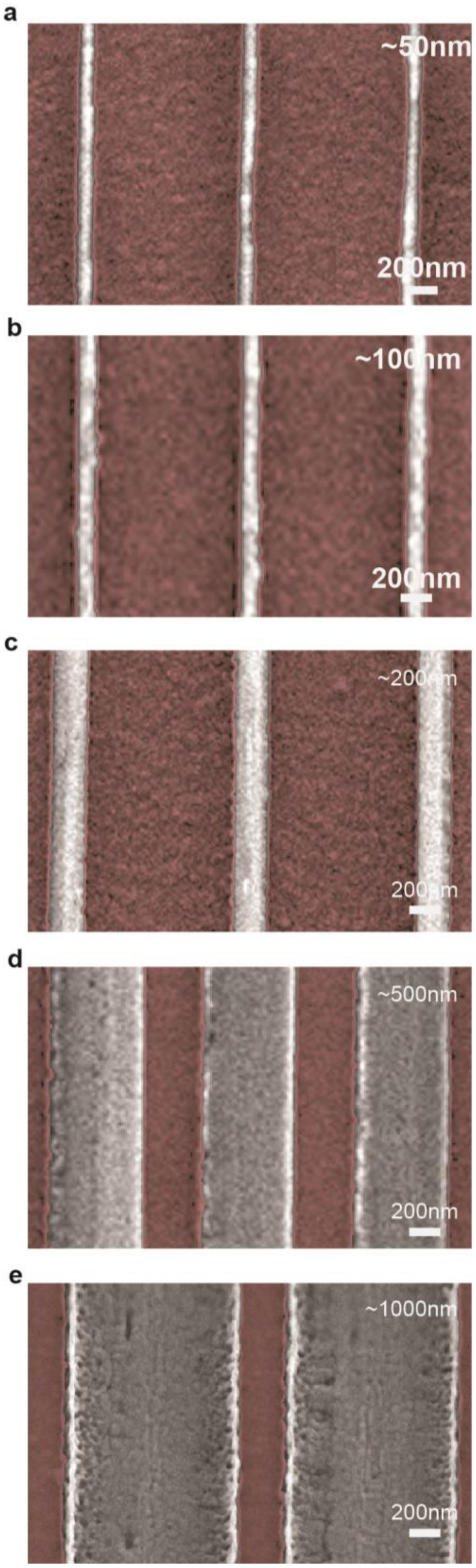

Fig. S1. Colored SEM micrographs of a series of $MAPbI_3$ nanowires (grey color) grown in ZEP520A channels (purple color) with altered periodicity and channel width. The channel width was set to 50, 100, 200, 500 and 1000 nm respectively. (a-e)

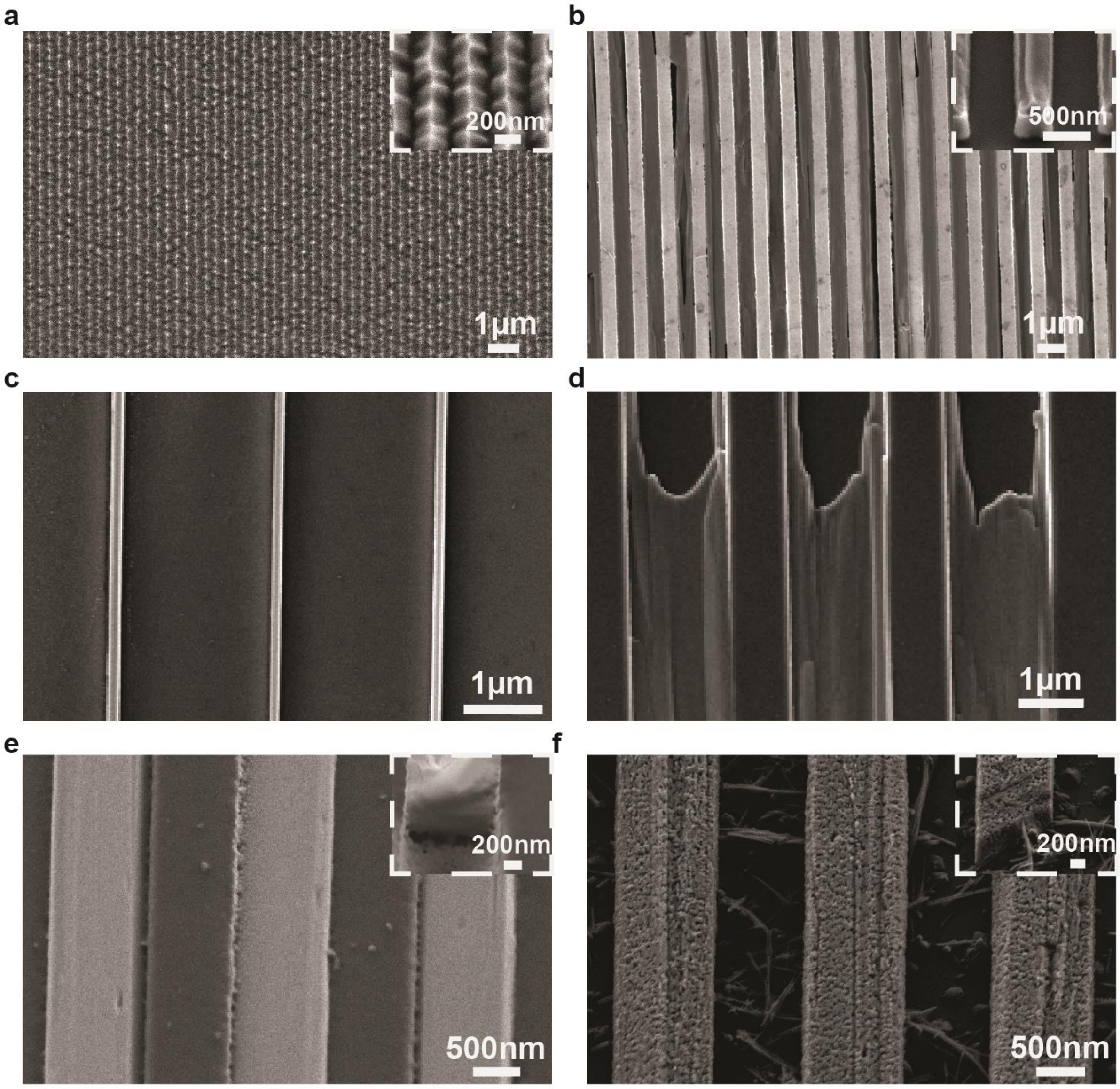

Fig. S2. SEM micrographs of a dense series of nanochannels with triangular (**a**) and rectangular (**b**) cross sections fabricated on ST-cut quartz. The perovskite nanowires fill the nanofabricated channels, adopting their shapes and aspect ratios. Because of the heterogeneous nucleation, larger channels have a higher probability of yielding bunches of nanowires (**d**) than narrower ones (**c**). The nanowires grow both on flat TiO_2 surfaces made by ALD (**e**) and rough substrates made by spin-coating TiO_2 nanowires on the ALD deposited TiO_2 (**f**).

EDX analysis

Scanning Electron Microscope images were acquired with a MERLIN Zeiss electron microscope. The elemental composition of the $MAPbI_3$ crystallites was analyzed by EDX. Samples were mounted on Al pucks with carbon. Energy-dispersive X-ray spectroscopy (EDX) measurements were done with an X-MAX EDS detector mounted at a 35 degree take-off angle with a SATW window. EDS spectra and map were obtained at a working distance of 8.7 mm with 10 keV accelerating voltage and a current held at 200 pA. 1024 channels were used for acquisition, corresponding to energy of 10 eV per channel. The EDS map was taken at a magnification of 5687x corresponding to a map area of 20.2x15.2 μm^2 with a resolution of 2048x1536 pixels. Spectra were acquired over 74 seconds of live time with detector dead time averaging of 4% and a dwell time per pixel of 10 µs. Quantitative EDS analysis utilized AZtec software provided by Oxford Instruments Ltd. The Pb:I ratio in the as-synthesized filiform nano crystallites and single crystals is found to be ~3, which is consistent with the formula of $MAPbI_3$.

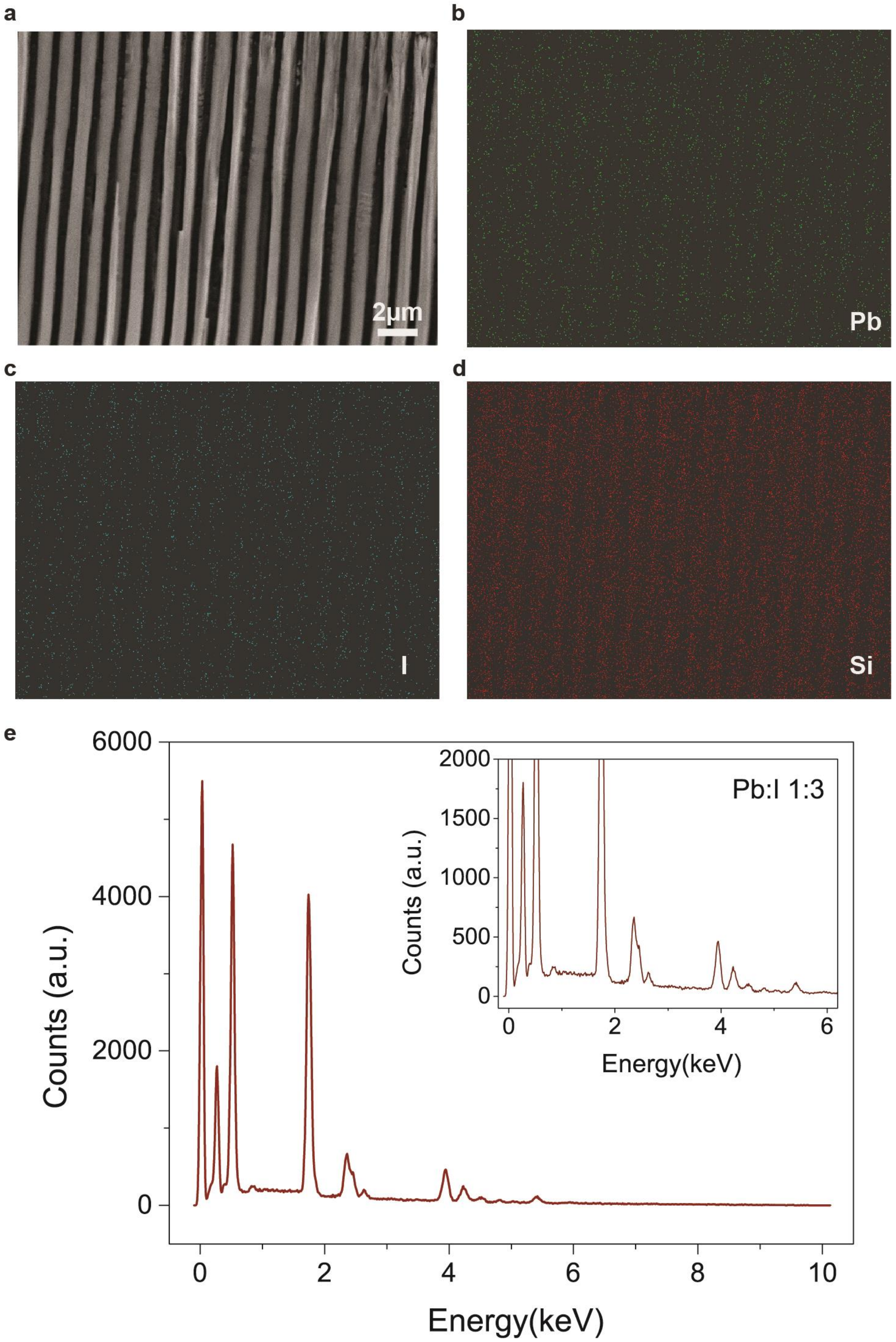

Fig. S3. SEM micrograph of set of $MAPbI_3$ nanowires synthesized in quartz nanogratings (**a**). EDX window integral maps of Pb (**b**), I (**c**) and Si (**d**). Note that the majority of signal characteristic to the elements of Pb and I were detected from the grooves filled with $MAPbI_3$ nanowires. The low-intensity signal detected on the

“uncovered area” is related to the presence of small isotropic $MAPbI_3$ crystallites generated from the solution overflow from the nanofluidic channels. EDX sum spectrum calculated from the data acquired from all the pixels in the secondary electron image (**e**).

Fluorescence Microscopy

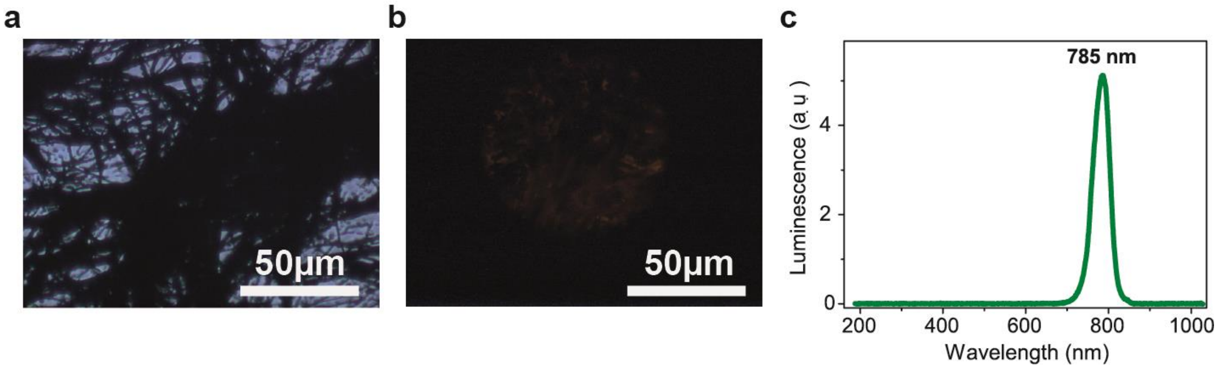

Fig. S4. The optical microscopy image of perovskite nanowire bundles (**a**), the corresponding fluorescence image obtained upon excitation with green monochromatic incoherent light at λ_{ex}=546 nm (**b**), and the photo-emission spectrum of perovskite nanowires acquired upon excitation at λ_{ex}=546 nm, having the peak intensity at 785 nm and matching well the characteristic emission of $MAPbI_3$ at ~1.58eV (**c**).

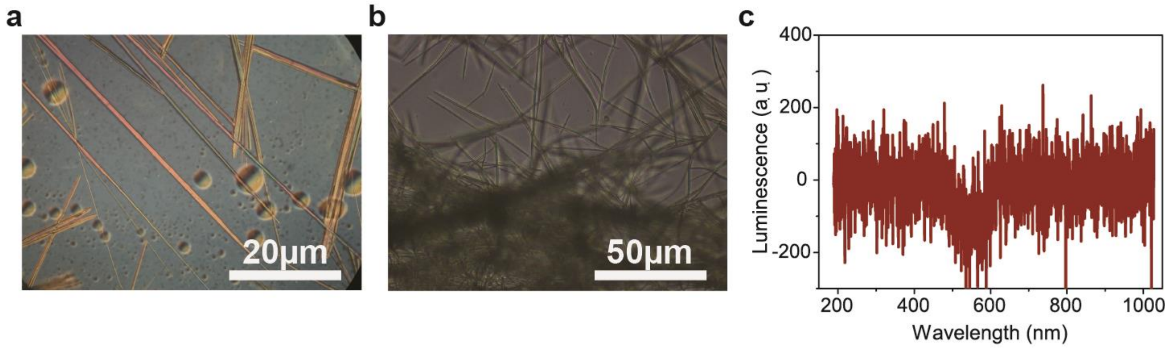

Fig. S5. The optical microscopy images of perovskite nanowire $MAPbI_3$-DMF bundles in solvatomorph phase with DMF (**a, b**). The corresponding photo-emission spectrum of perovskite nanowire solvatomorph acquired upon excitation with green monochromatic incoherent light at λ_{ex}=546 nm (**c**). Note the total absence of the characteristic photo-luminescence of $MAPbI_3$ (peeking around 785 nm) in the spectrum depicted in **c**.

$MAPbI_3$ nanowires dissolution

We conducted *in-situ* nanowire dissolution experiments. The nanowires for this study were prepared on a glass microscope slide by the standard slip-coating process. Here, we add excess of polar aprotic solvents (DMF, DMAc and DMSO) to dissolve the $MAPbI_3$ nanowires. The dissolution experiment revealed some fascinating behavior of $MAPbI_3$ nanowires etching in their mother liquor. As it can be seen in Fig S6 the dissolution of elongated $MAPbI_3$ is highly anisotropic. In contact with excess of DMF the dissolution starts and rapidly advances from both ends of the wires (the same planes as during growth). Only minor dissolution was observed on the sidewalls, the facets perpendicular to the growth facets. This suggests that trough careful control of the experimental conditions selective etching of perovskite nanowires is feasible.

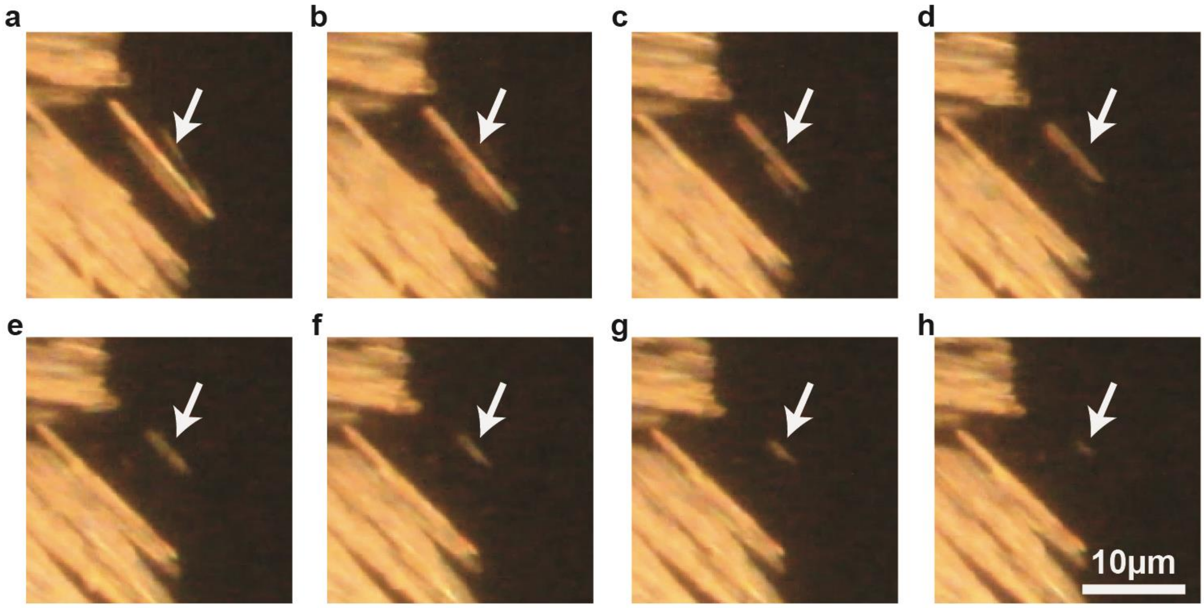

Fig. S6. Snapshots (**a-h**) from a video showing the dissolution of the $MAPbI_3$-DMF solvatomorph NWs in their mother liquor (DMF). The nanowire dissolution is highly anisotropic. The dissolution starts and rapidly advances from both ends of the wires. Only minor dissolution was observed on the facets perpendicular to the growth facets.

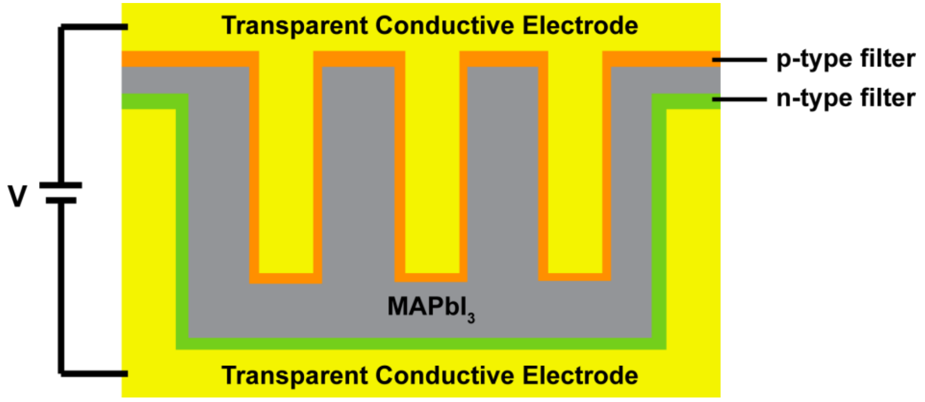

Fig. S7. Top view schematics of a conceptual interdigitated perovskite solar cell architecture. The planar heterostructure used nowadays could be replaced by an in interdigitated configuration where the higher surface-to-volume of the nanowires, their crystallinity and the improved charge collection efficiency due to the reduced distance of the electrodes could lead to higher solar-to-electric conversion efficiencies. Additionally, the absence of the top transparent conductive electrode would reduce the absorption/reflection losses.

Video S1. This video, recorded in an optical microscope with a Canon EOS 5D Mark III, shows the real-time growth of $MAPbI_3$ nanowires grown on a dense array of Si grooves. Each groove is 500 nm wide, as is their spacing. The array extends over 2 mm. Once the saturated $MAPbI_3$ solution is dropped onto the array, capillary forces drive the liquid inside the open nanofluidic channels.

Video S2. White light directed at different angles on the surface a Si chip containing series of $MAPbI_3$ nanowires grown in ZEP520A channels with altered periodicity and channel width. The ZEP520A was subsequently dissolved after the graphoepitaxial growth. The intense blue, green, orange and red colors are due to the interference pattern of millimeter long periodic perovskite nanowire arrays grown on the surface of a Si chip. The video was recorded with a 150 mm macro lens (Sigma 1:2.8 APO MACRO DC HSM) mounted on the body of a Canon EOS 5D Mark III full frame DSLR camera.

Video S3. This video, recorded in an optical microscope with a Canon EOS 5D Mark III, shows the growth of $MAPbI_3$ nanowires grown on a glass substrate covered with 100 nm thick gold coating. The most important parameter determining the nanowire diameter might be the size of the nucleation center. Once the nanowire growth starts, the cross-section remains constant over ~cm length scales. The video was speeded up 8 times.

Video S4. This video, recorded in an optical microscope with a Canon EOS 5D Mark III, shows the growth of $MAPbI_3$ nanowires grown on a glass substrate covered with 100 nm thick gold coating. This viedo shows the growth of nanowires grown from multiple nuclei resulting in the formation of crystallographically-fused parallel aggregates of perovskite nanowires. Once the nanowire growth starts, the cross-section remains constant over ~cm length scales The video was speeded up 8 times.
